# The rise of single‐cell proteomics

**DOI:** 10.1002/ansa.202000152

**Published:** 2021-02-01

**Authors:** Claudia Ctortecka, Karl Mechtler

**Affiliations:** ^1^ Research Institute of Molecular Pathology (IMP), Vienna BioCenter (VBC) Campus‐Vienna‐Biocenter 1 Vienna 1030 Austria; ^2^ Institute of Molecular Biotechnology of the Austrian Academy of Sciences (IMBA), Vienna BioCenter (VBC) Dr. Bohr‐Gasse 3 Vienna 1030 Austria; ^3^ Gregor Mendel Institute of Molecular Plant Biology, Austrian Academy of Sciences, Vienna BioCenter (VBC) Dr. Bohr‐Gasse 3 Vienna 1030 Austria

**Keywords:** mass spectrometry, single‐cell proteomics, ultra‐low input proteomics

## Abstract

Mass spectrometry‐based proteomics comprehensively defines proteome expression patterns in thousands of cells majorly contributing to our current understanding of many biological processes. More recently, single‐cell transcriptome and genome studies, however, have demonstrated overwhelming heterogeneity of tissues and cellular subpopulations. These studies have indicated different cellular functionality and identity, which are mainly driven by proteins and their posttranscriptional modifications. The rapidly emerging field of single‐cell proteomics aims at complementing transcriptome and genome data by generating comparative protein expression profiles from individual cells. Recent developments demonstrated tremendous improvements in sample preparation workflows and MS instrumentation, quantifying over 1000 proteins from a single cell. Efficient and reproducible sample processing in conjunction with sensitive MS acquisition strategies will allow to further increase the proteome coverage of tissues with single‐cell resolution. The required throughput and data reliability of such studies are still subject to further developments. Therefore, we herein discuss recent progress on specialized workflows and instrumentation next to advancements outside the field, which we expect to contribute to the development of comprehensive single‐cell proteomics.

## 1 INTRODUCTION

Single‐cell analysis aims at differentiating cell types and subpopulations originating from heterogeneous samples according to global expression differences. Comprehensive transcriptome based single‐cell studies demonstrated that heterogeneity is intrinsic to the nature of individual cells and arises from stochastic influences (i.e., microenvironment).^1^, ^2^ However, proteins and specifically their posttranslational regulation are the main driver of cell functionality and cellular identity. These patterns are subject to rapid changes and require direct protein measurements without the need to infer their function from mRNA measurements.^3^ Mass spectrometry (MS) very successfully allows for hypothesis‐free analysis of the proteome and various posttranslational modifications (PTMs) from bulk samples.^4^, ^5^, ^6^ Whether stochastic fluctuations have functional significance and if they represent biologically meaningful distinctions within or between populations requires identification of heterogeneity patterns with single‐cell resolution. This has largely been addressed by hypothesis‐driven methods such as fluorescent activated cell sorting (FACS), immunohistochemistry, and mass cytometry. While these studies majorly contributed to many fields, antibody‐based systems heavily rely on the specificity and availability of such probes, measuring up to 50 signals per experiment.^7^

**FIGURE 1:**
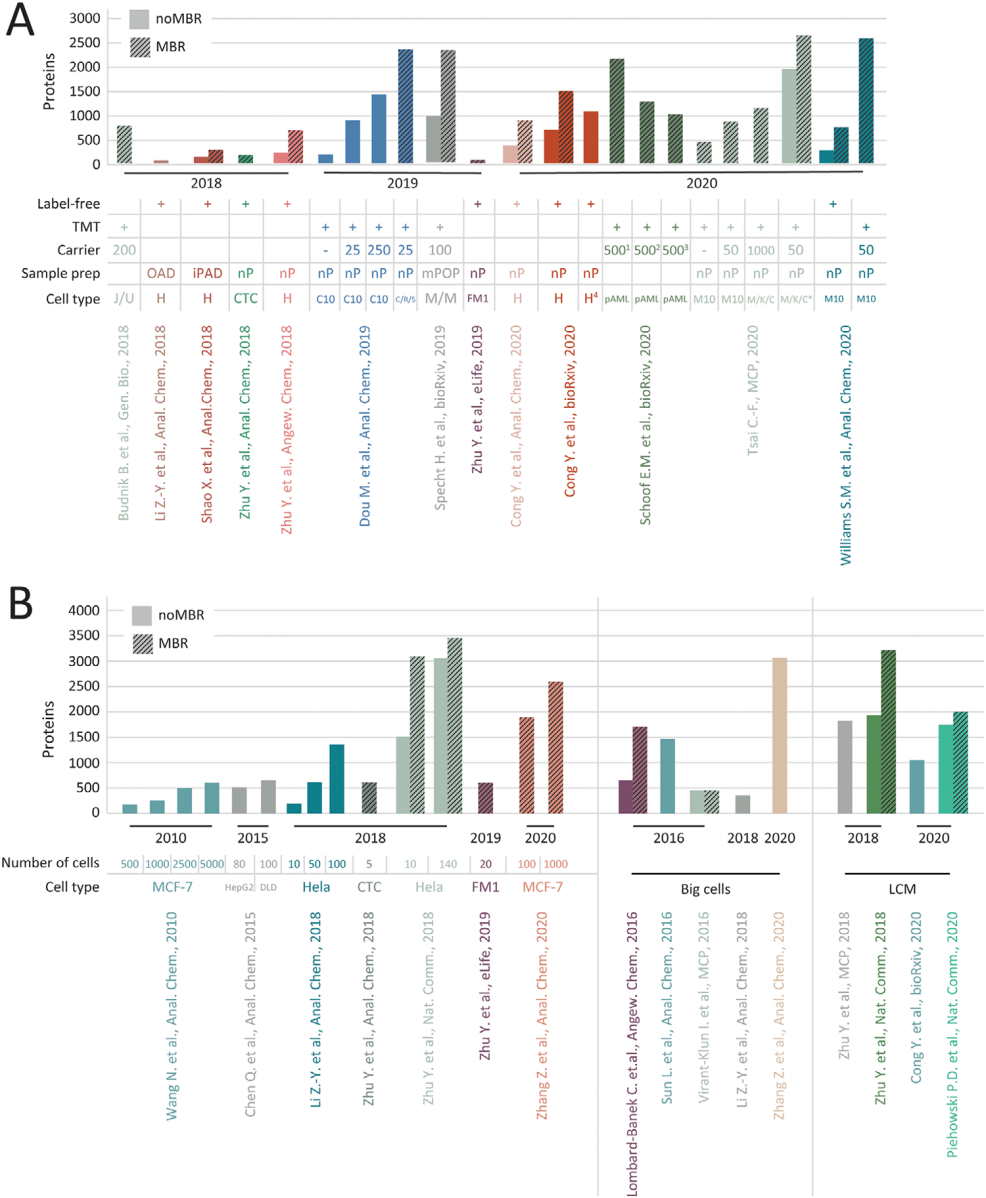
Overview of protein identifications (A) from single‐cell proteomics measurements including respective sample preparation technique, quantification method and cell type. B, Comparison of protein identifications from proteomics experiments utilizing less than 1000 cells along with the number of cells used in the study, cell type, and if obtained from tissue sections via LCM. Use of match between run is indicated. Protein identifications for (A) and B were obtained from the publications directly, search parameters might vary. J/U, Jurkat/U937 cells; H, HeLa; CTC, circulating tumor cells; nP, nanoPOTS; C10, C10 mouse cell line; C/R/S, C10/RAW/SVEC; M/M, monocytes to macrophage differentiation; FM1, FM1‐43low and FM1‐43 high labeled chicken utricle cells; pAML, primary amyloid leukemia hierarchy ‐ leukemic stem cells, progenitors and blasts; M10, MCF10A; M/K/C, MOLM‐14, K561, CMK; * ‐ 104 FACS‐sorted cells in 13 TMT‐plexes; 1 – bulk and cell type‐specific carrier; 2 – bulk carrier; 3 – cell type‐specific carrier; LCM, laser capture microdissection

The fast‐paced field of single‐cell proteomics aims at generating hypothesis‐free protein expression profiles from individual cells. Despite the tremendous technological improvements in ultra‐sensitive proteomics workflows and instrumentation, the comprehensive characterization of individual mammalian cells still challenges current MS‐based proteome analysis methods. The accentuated combination of developments in sample recovery, instrument sensitivity, and data analysis strategies will promote their analytical performance and achieve higher proteome coverage. Early reports of such MS‐based single‐cell proteomic analysis demonstrated in‐depth characterization of large cells, such as oocytes and blastocytes with 700 ng of protein (Figure 1B).^8^, ^9^, ^10^ With major advancements in instrumentation and sample preparation workflows, the first mammalian single‐cell measurements were published in 2018, using the combination of isobaric‐labeled single cells with a highly abundant congruent carrier sample.^11^ Rapidly increasing numbers of publications within the past years address diverse technological bottlenecks and demonstrate biological applicability for several cell types, micro‐dissected tissues, and sorted sub‐populations (Figure 1A).^12^, ^13^, ^14^, ^15^ The quantification of more than 1000 proteins was described using specialized sample preparation workflows and dedicated acquisition strategies in label‐free and multiplexed experiments (Figure 1A).^13^, ^16^

The successful rise of single‐cell proteomics is a multidisciplinary effort including miniaturized sample preparations, optimized chromatographic separation, sensitive MS instrumentation with dedicated acquisition strategies, and refined bioinformatics approaches. We hereby want to focus specifically on technological progress, acquisition strategies, and postprocessing approaches that recently demonstrated advancements to the field. We aim to critically evaluate current techniques, illustrate improvements, showcase opportunities, and address limitations of current single‐cell proteomics studies.

## 2 SAMPLE PREPARATION

Standard bottom‐up proteomics encompasses a multistep procedure with cell extraction and lysis, protein extraction and solubilization, reduction of disulfide bonds and alkylation of cysteines, digestion of proteins to peptides, sample clean‐up followed by chromatographic separation and MS acquisition. These processes have been optimized for micrograms of sample input in which anticipated losses do not impair subsequent analysis. In single‐cell proteomics, however, given that one cell contains around 200 picograms of protein it is crucial to minimize losses during sample preparation.

### 2.1 Adsorptive losses

Outstanding robustness down to several thousand Hela cells was demonstrated with the popular filter‐aided sample preparation (FASP) technique, processing samples on commercial spin filters. Reduction and alkylation, digestion, and elution of peptides into MS‐compatible solvents are performed within the same molecular weight filter unit.^17^ More recently, this method was miniaturized to a pipet tip format (micro‐FASP) reducing the sample elution volume from over 100 µL to only 3 µL. Zhang et al recovered 3000 proteins from 1000 mammalian cells within one single analytical run using the micro‐FASP technique (Figure 1B).^18^ The drastic reduction in filter bed, preparation volumes, and sample handling steps minimizes exposed surfaces and therefore nonspecific adsorptive losses. The impact of multiple sample transfer steps were demonstrated with over 56% of peptide losses at 2 µg sample input.^19^ Nonspecific adsorptive peptide losses at such magnitude become more critical in single‐cell sample preparation. Reducing sample volumes and exposed surfaces or preconditioning of such surfaces is therefore regarded as essential.^20^, ^21^, ^22^

### 2.2 Protein solubilization

FASP not only reduces sample manipulation steps but the filter unit separates high molecular weight components (ie, proteins) from low molecular weight impurities and MS‐incompatible detergents. Strong detergents (e.g., sodium dodecyl sulfate, SDS) or extraction buffers (e.g., urea) facilitate robust protein solubilization, which remains a challenge in MS sample preparation workflows. Conventional ionic detergents disrupt tertiary structures of proteins and prevent protein aggregation, but suppress ionization and interfere with chromatographic separation, therefore are not compatible with LC‐MS analysis. Nonionic detergents are not as efficient in protein solubilization, however, some detergents such as *n*‐dodecyl‐B‐d‐maltoside (DDM) are MS compatible at low concentrations. Acid degradable detergents have been developed for proteomics workflows as they exhibit their full potential during sample preparation but degrade into innocuous by‐products prior to MS analysis.^17^, ^23^, ^24^ Detergents are highly efficient in cell lysis and protein solubilization but often require buffer exchanges prone to peptide losses, biases, and introduction of contaminants. Previously mentioned filter‐aided sample preparation techniques combine buffer exchanges and sample cleanup steps with the sample preparation.

### 2.3 Reduced sample manipulation

Advancing from the FASP protocol, C_18_ discs regularly used for sample clean‐up prior to MS analysis were inserted into pipet tips (i.e., StageTips) to create reaction tubes. With this approach strong detergents are avoided in contrast to the molecular weight filters used in FASP. The in‐StageTip (iST) protocol allows for simultaneous lysis, protein solubilization, reduction, alkylation, and enzymatic digestion. Complete sample preparation is performed in one reaction vessel with fewer pipetting steps, reduced sample volumes, and removal of impurities prior to peptide elution.^25^ Combined peptide purification and separation using StageTips were recently automated using “preformed gradients” and “offset gradients for peptide refocusing” implemented in the Evosep One platform. Digested samples are loaded to a disposable trap column (C_18_ StageTip or EvoTip™) and eluted with increasing organic content. A secondary gradient at the head of the tip reduces the organic content of this preformed gradient, shortly retaining the eluting peptides and therefore sharpening peak widths. These refocused peptides are then switched in‐line and loaded onto the analytical column.^26^ The automation of peptide purification coupled to chromatographic separation reduces error‐prone manual handling steps and limits exposed surface areas. The disposable Evotip™, however, is not saturated prior to sample processing, increasing adsorptive losses to the column bed. Nevertheless, ultrasensitive single‐cell analysis has been proposed as one of Evosep One's specialized applications in combination with the tims‐TOF Pro.

### 2.4 Designated designs

Miniaturization and sample handling simplification were successfully combined for the development of the nanodroplet processing in one pot for trace samples (nanoPOTS) by the group of Ryan Kelly. The nanoPOTS are glass chips microfabricated with photolithographically patterned hydrophilic pedestals surrounded by a hydrophobic surface serving as nanodroplet reaction vessels. A glass spacer is sealed to a coated glass slide to minimize evaporation within the nanoPOTS. Their initial sample preparation protocol incorporates simultaneous lysis, protein denaturation, and reduction using, RapiGest, an acid‐labile detergent with the reducing agent Dithiothreitol. Followed by alkylation of cysteines and protein digestion with LysC and trypsin. Their homebuilt robotic platform facilitates pico‐liter dispensing for a final sample volume of only 200 nL. Samples are then collected in a fused silica capillary, sealed with parafilm, and stored in a freezer until MS analysis. They initially demonstrated comparable digestion efficiency to bulk samples and identified 1517 proteins from only ten mammalian cells (Figure 1A).^27^ nanoPOTS demonstrate that reduction in sample volume and minimizing exposed surface areas is pivotal for sample recovery and achieves appropriate ratios between chemicals to the sample. Lysis and digestion conditions (i.e., enzyme to substrate ratio) optimized for bulk samples must be adapted to the extremely reduced protein concentration of single cells to ensure effective chemical lysis, improved digestion efficiency, and reduced autolysis.^28^, ^29^

Since the initial publication, the nanoPOTS platform was applied to various other samples such as laser micro‐dissected tissue of 10‐18 cells which identified 1000 proteins (^30^ ^,^ ^31^), 670 protein groups from single Hela cells,^27^ 160 protein groups from circulating tumor cells,^32^ 200 unique peptides from sensory hair cells during chicken development^15^ and most recently the differentiation of two neural sub‐types with over 1000 proteins identified from a single cell^16^ (Figure 1A & B). In one of their latest laser capture microdissection studies, a quantitative, spatially resolved and cell‐type specific protein map was established with 100 µm resolution identifying around 1500 proteins per field (Figure 1B).^33^ The combination of nanoPOTS with a popular carrier method using isobaric labels identified up to 1200 proteins per single cell (Figure 1A)^34^ (carrier method is discussed in ‘4. Quantification’).

By applying a more minimalistic approach completely avoiding detergents, chaotropic agents, and other chemicals, Specht et al. use standard laboratory equipment for sorting cells into pure water using a common 384‐well plate. Lysis is performed with extreme freeze and heat cycles (‐80°C / 95°C), immediately followed by tryptic digestion for 4 hours in a thermocycler for limited evaporation.^35^ Due to the microliter sample volume, a dramatic increase in enzyme concentration is required, in turn elevating the risk of chymotryptic activity or interfering with the analysis.^28^, ^29^ After digestion, the samples are labeled with standard isobaric labels including an abundant congruent carrier sample,^35^ which is detailed in ‘4. Quantification’.

Efficient transfer of the sample to the HPLC remains the missing link between sample processing and MS analysis. This was first approached with the oil‐air‐droplet (OAD) chip, in which nanoliter volume sample processing is performed in small reactors sealed with a layer of oil. This oil seal can be penetrated with a capillary nanopipette and automatically re‐seals the reaction chamber without direct oil‐sample contact. Using a high‐pressure pneumatic pump the sample is directly loaded to the chromatographic column, identifying 51 proteins from 1 Hela cell (Figure 1A&B).^36^ Similarly, a custom autosampler for nanoliter samples enabled automatized injection from the nanoPOTS. Processed samples are dried within the nanoPOTS using a desiccator and then directly loaded to an SPE column via the home‐built autosampler (Figure 1A).^37^ Manual transfer and injection of single‐cell samples using standard autosamplers is error prone and subject to substantial losses when handled inappropriately. With the integrated proteome analysis device (iPAD) these manual steps were combined into the processing workflow. Sample preparation is performed in a capillary, which can be directly connected to the analytical column. Single cells, a chaotropic agent and trypsin are aspirated in 2 nL with air gaps between the samples to ensure separated reaction vessels. Samples are sonicated at 50°C for enhanced lysis and digestion followed by chromatographic separation and MS analysis (Figure 1A & B).^38^, ^39^

Multiple groups independently address single‐cell sample preparation by adapting steps of the standard proteomics workflows, reducing sample volumes and improving kinetics, however, a universal and robust protocol is still lacking. Sample miniaturization and automatization, as demonstrated by several research groups will pave the way for future technical developments, enhanced sample recovery and reproducible single‐cell analysis. The promising nanoPOTS platform currently requires a customized liquid handling robot operated by skilled personnel. We are confident that as soon as a workflow can be implemented in more laboratories without the need for personalized instrumentation, diverse expertise will rapidly advance single‐cell proteomics sample processing workflows.

## 3 CHROMATOGRAPHIC SEPARATION

Designated sample preparation and optimization of chromatographic support materials for improved separation and peak capacities are closely associated. In this review we only briefly touch upon the topic of chromatography and ionization for single‐cell proteomics, which have been extensively discussed elsewhere.^29^, ^40^ We still want to highlight the importance of lowering flow rates to nanoliters per minute, improving peptide ionization by electrospray and thus sensitivity.^41^, ^42^, ^43^ Additionally, narrow‐bore columns generate sharper peaks with higher analyte concentrations and consequently improved signal intensity. The superior peak width at comparable flow rates of monolithic or etched columns, in contrast to slurry packed columns is ideal for ultra‐low input chromatography.^44^

### 3.1 µPAC

Ordered micropillar structures rather than a disorder slurry for separation were commercialized by PharmaFluidics. Our group demonstrated doubling of unique peptide and protein group identifications when using µPAC compared to standard C_18_ PepMap columns. We speculate that the core‐shell architecture of µPAC micropillar structure reduces losses and increases peptide precursor signals. Additionally, the full width half maximum is strongly reduced and the µPAC columns show an unprecedented degree of retention time (RT) stability.^45^ The current design outperforms slurry packed columns, however, with more narrow pillars or a reduced inner diameter (i.d.) these could improve further for ultra‐low input samples.

### 3.2 Narrow‐bore columns

Flow rates of 20 nl per minute were recently demonstrated by decreasing chromatographic column i.d. from standard 75 µm to 20 µm. This slurry packed column is fused to an etched silica emitter and directly connected to a SPE column pre‐loaded with the sample for decreased overhead times. The reduced bore size results in improved ionization efficiency, greater ion flux with less solvent‐associated chemical noise and 20% more protein identifications from ultra‐low samples (i.e. 362 proteins from single Hela cell – Figure 1A).^46^ More recently a 2 µm i.d. column in an open tubular format with 790 pl per minute flow rate demonstrated remarkable sensitivity. However, the column requires nitrogen sheath flow to stabilize the electrospray stability and minimize chemical background noise to retrieve peptide identifications.^47^ These and many other studies demonstrate the still unexploited potential of chromatographic separation material and its essential contribution to the analysis of single‐cell samples.

## 4 QUANTIFICATION

Next to the developments in miniaturized sample preparation technologies, the use of multiplexing techniques by *in vitro* stable‐isotope labeling of peptides (e.g. Tandem‐Mass‐Tag, TMT) have been identified key to the comparative analysis of protein expression levels in individual mammalian cells by MS. Label‐free quantification in combination with nanoPOTS was demonstrated highly successful in studying various cell‐types with single‐cell resolution (^27^, ^32^, ^37^ ^,^ ^46^). However, stochasticity of precursor sampling increases missing data of label‐free analysis in large cohorts^48^ compared to isobaric labeled samples. Stable‐isotope labeling of peptides with isobaric tags is a commonly used technique in standard proteomics workflows to simultaneously and relatively quantify up to 16 samples at increased throughput.^49^ Isobaric tags like TMT have an identical total mass while heavy isotopes are distributed differently across the tag. Thus, peptides from all combined samples co‐elute during chromatographic separation and are selected as one precursor. Fragmentation generates reporter ions with different isotopic loading of each tag and therefore a unique quantitative peak for each sample at a defined m/z. These multiplexing capabilities increase the number of samples analyzed within a single analytical run 16‐fold, consequently elevating low abundant peptides above the lower limit of detection. Most importantly, however, the relative quantification between multiple samples vastly reduces missing data compared to label‐free analysis in data dependent acquisition (DDA).

TMT multiplexed studies have been increasingly popular in the analysis of ultra‐low input or single‐cell samples. In recently proposed workflows for Single Cell ProtEomics by Mass Spectrometry (SCoPE‐MS, Figure 1A,^11^) the analysis of 8 individual single cells is multiplexed with highly abundant congruent carrier material (i.e. derived from 200 cells) in one analytical run. SCoPE‐MS combines isobaric labeled carrier peptides with single‐cell samples in order to reduce sample loss, increase abundance of precursor ions and serve fragment ions for peptide identification.^13^ The highly abundant carrier increases the signal of the single cells during MS1 scans, accordingly, improving the signal to noise ratio (S/N) of the isolated precursor. This improved S/N in MS1 scans allows for more efficient triggering of peptides of interest. Additionally, the carrier improves peptide identification by increasing the number of ions sampled. Importantly, however, due to well‐described effects such as inter‐channel ratio compression^50^, ^51^, ^52^, ^53^, ^54^, ^55^ the relative quantitation of such extremely imbalanced TMT‐reporter ion signal intensities is widely considered as being particularly error prone.^56^, ^57^

The SCoPE2 workflow partially addressed the extremely abundant carrier sample, by reducing its ratio from 200 to 100 cells and introducing a reference channel for relative quantification of the single‐cell channels within multiple TMT batches.^13^ Exemplified on their SCoPE2 workflow, they demonstrated more accurate quantification with increased AGC targets.^58^ The thereby prolonged cycle time dramatically reduces the number of MS/MS scans and peptide identifications accordingly. The combination of single cells with a highly abundant carrier sample overcomes losses during chromatographic separation and improves ion counts. However, the ‘carrier proteome effect’ has been recently evaluated in great detail, demonstrating that carrier protein amounts above 100x impact quantitative accuracy.^59^ With higher carrier sample amounts, increasing numbers of ions are required to maintain quantitative accuracy.^53^, ^60^ However, extensive sampling of ions in combination with high carrier proteomes (i.e. > 75x) may lead to underestimation of multiplexed single‐cell samples. Further expanding the number of multiplexed samples (i.e. 6‐plex to 8‐plex) also requires more ions for similar quantitative accuracy.^59^, ^61^ A carefully evaluated balance of carrier amounts and ion counts is necessary for accurate ratio reporting. SCP Companion conveniently combines quality control, instrument parameter evaluation and carrier ratio estimation into one tool. These recent insights strongly highlighted that careful selection of acquisition parameters, drastic reduction of the carrier to a minimum (i.e. 20x) and post‐acquisition S/N filtering can improve biological conclusions from such experiments.^59^

So far SCoPE has been applied to differentiating embryonic stem cells,^11^ macrophage heterogeneity,^13^ acute myeloid leukemia cell lines in combination with nanoPOTS^62^ and differentiating leukemic stem cells^12^ (Figure 1A). In the latter, different carrier compositions, including a cell‐type specific, a bulk or a combination of both were investigated. It was demonstrated that by PCA analysis only a cell‐type specific carrier allows for successful clustering of the single cells. Randomized cells within TMT batches only cluster according to their analytical runs.^12^ The carrier sample determines the selection of precursors and mainly contributes ions for peptide identification. Consequently, the selection of appropriate carrier cells for each experiment and across multiple analytical runs is crucial for cell type specific clustering.

Similarly, carrier samples have been used for the semi‐targeted analysis of phosphorylated peptides using an IMAC enriched carrier channel in combination with lower‐input patient samples to boost phospho‐peptide identification two‐fold.^63^ The combination with a label‐free carrier protein (i.e. BSA) for off‐line fractionation, as previously demonstrated,^21^, ^64^ further improved their phospho‐peptide identifications two‐fold. Carrier molecules have been extensively applied in great variety, yet the conjoint analysis of such extremely imbalanced multiplexed samples, especially for the analysis of single‐cell samples remains debated. Improving the analysis depth of single‐cell proteome analysis but compromising quantitative accuracy still leaves room for advancements. We hypothesize that with developments of more sensitive MS instrumentation, the necessity of such extreme carrier samples for single‐cell proteomics can be overcome.

## 5 MS INSTRUMENTATION

Improvements in sample preparation, chromatographic separation and more efficient and sensitive MS instrumentation accentuate successful single‐cell MS measurements. Instrument parameters require careful evaluation and adaptation to extremely low ion counts. Ultra‐low samples necessitate long inject times to accumulate sufficient ions for accurate quantification or peptide identification. This, however, results in extended duty cycles, inefficient usage of the ion flux and focus only on the most abundant precursors. We expect that acquisition techniques such as data independent acquisition (DIA) and BoxCar addressing the intra‐scan dynamic range (highest and lowest signal intensities observable within the same MS/MS scan) will refocus on low abundant precursor signals to improve ultra‐low sample analysis.^65^, ^66^

### 5.1 Trapped ion mobility spectrometry (tims)

Similarly, trapped ion mobility in conjunction with parallel accumulation‐serial fragmentation (PASEF) on the recently developed tims‐TOF Pro synchronizes precursor selection, filters multiply charged precursors and separates co‐eluting peptides by their collisional cross‐section. Briefly, as ions are injected into the MS they are trapped within the tims funnels by a continuous gas flow and an opposing electrical field. While stepwise eluting ions into the quadrupole from the second tims device, newly arriving ions are simultaneously trapped in the first. Using the PASEF acquisition strategy a 50 ms ion mobility scan is accumulated for topN determination and sub‐millisecond quadrupole switching allows to select multiple precursors for fragmentation. Due to ion mobility separation the noise remains distributed but precursor signals are compressed in a shorter time, consequently improving S/N.^67^ The increased sequencing speed without compromising sensitivity may result in enhanced reproducibility between ultra‐low samples. MS based analysis of single‐cell samples is inherently prone to overwhelming amounts of missing data and low reproducibility of multiple analytical runs. This has been mainly addressed computationally, as described in ‘6. Post‐processing and data analysis’, however we speculate that improving reproducibility, diminished by the stochasticity of precursor picking in DDA, is key to reliably study single cells at large scale.

### 5.2 Data independent acquisition (DIA)

This acquisition type is defined by efficient use of the ion flux aiming at isolating and fragmenting all precursor populations within a defined mass range by cycling through predefined m/z segments. To this end, the speed and ability of summing ion intensities from several transitions for enhanced S/N and more accurate measurements in TOF instruments has been made use of. In the first reports of such acquisition modes with widened isolation windows of 10 Da, did not impact the overall noise level on peptide identifications. The decreased cycle time resulted in about 20% more MS/MS spectra, however, this did not translate into more peptide or protein identifications. When using such large isolation windows, chances of isolating several precursors at once ( = chimeric spectra) increases, which are inherently difficult to identify. Additionally, co‐isolation of highly and lowly abundant precursors may bias peptide identifications towards the higher abundant fragment ions.^68^ This method was extended with SWATH‐MS (Sequential Window Acquisition of All Theoretical Mass Spectra) further increasing isolation windows to 25 Da and decreasing the selected mass range. Additionally, precursor isolation windows overlap to ensure complete transfer of the isotopic pattern in at least one of the transitions. The limit of detection of SWATH‐MS is in the amol range with an intra‐scan dynamic range up to 4 orders of magnitude.^65^ The introduction of RT standards and optimal resolution of the chromatographic peak with 2.3 seconds cycle time allows for reproducible, precise and comprehensive MS based proteomics studies of large cohorts.^69^ This was recently demonstrated by the acquisition of 1560 analytical runs in DIA mode on several QTOF instruments. Several factors possibly impeding DIA processing such as technical noise, LC maintenance, instrument calibration and RT shifts were evaluated, concluding that shorter gradients or small sample quantities might be most effective.^70^ Such short gradients (i.e. 15 minutes) were shown to be highly accurate for high‐throughput phospho‐proteomics compared to DDA. DIA analysis of low abundant PTMs improved the dynamic range by an order of magnitude with higher reproducibility across samples. This study was performed on a Q Exactive™ HF‐X hybrid quadrupole‐Orbitrap™ mass spectrometer and the authors hypothesize that with the ion usage of the tims‐TOF Pro, reproducibility could be substantially improved.^71^

### 5.3 diaPASEF

More recently the PASEF strategy was extended to a data independent mode. Standard DIA methods with a cycle time of about 2 seconds sample only about 5% of the total ion beam. In diaPASEF almost 100% of the ion signal is used in low complexity samples through the correlation of molecular weight and ion mobility on the tims‐TOF Pro. The extremely fast cycle time could be a major advantage for short gradients increasing throughput while sampling all precursors. Additionally, the reduced mobility of singly charged ions separates them from multiply charged in the mobilogram. Therefore, isolation windows along the ion mobility separated species allow for a selection of only multiply charged precursors for DIA analysis.^72^ We expect that the reported 89% data completeness of triplicate 10 ng Hela injections in diaPASEF to improve single‐cell proteomics analysis in large cohorts.

### 5.4 Targeted MS

Increased reproducibility and decreased missing data between large sample cohorts was also addressed by an improved targeted analysis method. Standard targeted MS methods are low throughput due to large RT windows, considering the expected RT variation between analytical runs. The combination with internal standards to adapt RT shifts between samples or trigger specific targets upon appearance of one or more standards has reduced RT windows and increased throughput of such methods.^73^ Using unspecific matrix molecules as RT alignment standards with one MS1 survey or 32 DIA alignment scans (cycle time 0.5 seconds) suffice to adjust RT shifts between individual samples on the fly. The recalibration of peptide inclusion lists allow to reduce RT windows and increase the number of targets within one analytical run three‐ to five‐fold.^74^

Many MS vendors have designated efforts towards single‐cell profiling for improved ion transmission and instrument sensitivity. Outstanding results were achieved with the latest Orbitrap Tribrid instrument in combination with nanoPOTS, a 20 µm narrow bore column described earlier and FAIMS, a high‐field asymmetric ion mobility device resulting in more than 1000 protein groups from a single cell.^16^ The FAIMS interface improves selectivity by filtering for multiply charged precursors, consequently improving the S/N, which is especially critical for low input samples as described above. Differential compensation voltages (CV) within FAIMS control the separation of ions and can improve proteome coverage.^75^ Cong et al. analyzed two post mortem neuron types with a dual CV method demonstrating great reproducibility and enhanced analysis depth (i.e. 2‐fold increase in peptide and protein identifications compared to no FAIMS).^16^ The latest generation Orbitrap instruments now enable on the fly real time search for enhanced precursor selection increasing peptide identifications by 14% for MS2 based TMT methods.^76^ In‐depth comparison of label‐free and multiplexed experiments showed that with current methods it remains challenging to rapidly, reproducibly, accurately, and sensitively detect and quantify large fractions of the proteome across multiple samples. TMT multiplexed workflows allow for higher throughput and identify 15 to 20% more peptides with higher quantitative precision compared to DIA. DIA, however, allows for short gradients, unprecedented reproducibility and better quantitative accuracy.^77^ This is of critical importance as single‐cell studies require the analysis of extremely large cohorts^78^ and multi batch TMT analysis remains challenging.^79^ DIA with short gradients and TMT‐multiplexed methods both result in high proteome coverage and low missing values in standard bulk analysis,^77^ which remains to be evaluated for single‐cell samples. Developments in MS instrumentation have been pivotal to current advances in single‐cell proteomics studies. However, tightly orchestrated instrument settings with the selection of appropriate acquisition modes remain demanding and subject to further investigation.

## 6 POST‐PROCESSING AND DATA ANALYSIS

Post processing and data analysis is the final but highly important aspect of MS analysis, as suboptimal choices may mask biological significance. Specialized data analysis strategies or a combination thereof can maximize the information obtained from such minimal samples but remain to be critically evaluated and controlled. Designated post processing tools for the growing field of single‐cell proteomics are still scarce, we however wanted to address current approaches and their applicability to such samples.

### 6.1 Spectral libraries

Compelling improvements in identification of DIA spectra were achieved through peptide centric analysis using spectral library matching.^80^ These libraries can be generated from published repositories or project specifically, the latter involving extensive additional measurement time but outperforming large resource libraries. Employing such libraries DIA identifies twice as many peptides compared to DDA with high coverage, reproducibility and precision.^69^ Three main approaches were designed to overcome the costly generation of project specific libraries. Firstly, *in‐silico* generated spectral libraries for enriched analysis of both DIA and DDA data are increasingly popular. Two approaches demonstrated the feasibility to compute such libraries based on neuronal networks from millions of tandem mass spectra. These tools allow to accurately predict fragmentation patterns and RT to enhance identification rates of fourier transform and ion‐trap MS analyzers, with varying charge states, peptide lengths, tryptic and non‐tryptic peptides.^81^, ^82^ Secondly, spectrum centric or library‐free DIA analysis outperforms resource and project specific libraries. DIA‐NN performs both, searches with a spectral library or library free, based on a protein sequence database,^83^ similarly to directDIA™ via Pulsar implemented in Spectronaut™. Thirdly, empirically corrected peptide predictions enable the fast generation of project specific spectral libraries for DIA. Gas phase fractionated samples are used to empirically correct fragmentation patterns and RT predictions to the current instrument status. Here the same sample matrix is used in the analysis and the library generation, in contrast to commonly used pre‐fractionated samples. The smaller theoretical search space outperforms *in silico* generated libraries due to better false discovery rate (FDR) calculations.^84^ Spectral libraries enhance identification rates and can maximize biological information obtained from proteomics samples, especially at low‐input, however missing data remains inherent and cumulative the more analytical runs are aggregated.

### 6.2 Missing data

Here we discuss three main types of missing data extensively reviewed in Karpievitch et al. (1) The peptide is present in the sample but not detected or identified, (2) the peptide is below the limit of detection or (3) the peptide is missing. Those are distinguished between missing completely at random (MCAR – type 1) and abundance‐dependent missing or censored values (type 2 and 3). MS data comprises both categories of missing data, challenging the selection of appropriate imputation and normalization methods. MCAR values often originate from ionization issues or interfering peptides, which can be imputed from a normally distributed empirical probability distribution. However, such simple imputation methods and most batch effect corrections are based on the distribution of all intensities. Here it is assumed that most peptide abundances do not change between samples and are therefore not appropriate for type 2 or 3. If data imputation is necessary, it should be performed prior to normalization to not computationally remove biological differences of the data. Nevertheless, the consecutive processing results in higher errors, as the second process does not consider the error of the first method. To avoid overfitting, differential expression analysis should always be controlled by their p‐value distribution.^85^, ^86^ DDA data of large cohorts often exceed missing values of 50%, with such large fractions of missing data most imputation mechanisms such as k‐nearest neighbors or mean imputation perform poorly. Even though many tools provide filtering parameters, it is not sufficient to prevent imputations from hiding inestimable contrasts and therefore should only be used if absolutely necessary. More advanced methods, that model the missing data might overcome some of the pitfalls in standard imputation, but it remains difficult to control the impact on the overall data.^48^

Isobaric label experiments can partially overcome missingness attributed to stochastic precursor selection or biases in RT. In multiplexed experiments, conditions are relatively quantified within one experiment, however when the samples exceed the number of available channels, multi‐batch TMT experiments have to be performed. In single‐cell proteomics, the analysis of only 16 cells (current maximum available number of isobaric labels) is by far not sufficient, multi‐batch TMT experiments are therefore the rule not the exception. Already when analyzing bulk samples one multiplexed experiment only contains 0.19% missing values at the protein level, but this readily increases to 6% upon data integration of a second run. This was exacerbated when 24 ten‐plex experiments were combined and only 6% of all peptides were identified in all experiments. This increase of missing data is not necessarily dependent on abundance of the peptides, but a combination of several factors discussed earlier. A common reference channel could reduce stochastic sampling effects and normalize batch effects.^79^ Therefore, the extremely abundant carrier channel of the previously described SCoPE strategy may improve precursor sampling in multi‐batch TMT experiments but was shown to be counterproductive for ion statistics and quantitative accuracy.^52^, ^59^, ^79^

### 6.3 Match between runs

One of the most frequently used approaches to address missing data in multi‐experiment analysis is match between runs (MBR). It transfers a peptide that is identified in one analytical run to another, in which the same peptide exists as MS1 feature within a defined RT tolerance, but was not selected for fragmentation or not identified by the search engine.^87^ Standard MBR can increase identifications but is performed after FDR filtering and is therefore not quality controlled. A two proteome experiment demonstrated falsely transferred yeast identifications to human‐only samples on average increases 8‐fold at the protein level when allowing MBR between 40 analytical runs.^88^ MBR was recently extended from label‐free analysis to isobaric labeled experiments and is integrated in the newest MaxQuant version. Isobaric MBR transfers identifications after recalibration of mass and RT via 3D MS1 features, to MS/MS spectra that were previously not identified and then uses their reporter ion intensities for quantification.^89^ MBR is exceptionally effective in boosting identification numbers and overcome stochastic sampling in DDA. However, it is highly error prone and difficult to estimate the real FDR of a dataset after MBR was performed. The latter was recently addressed by the development of IonQuant, a mixture model‐based approach to estimate the FDR of label‐free MBR. Exemplified on several publicly available datasets higher quantification precision, accuracy and sensitivity was demonstrated compared to the standard MBR integrated in MaxQuant.^90^ We are convinced, that this will increase confidence in feature matching and partially overcome the critical amounts of missing data in single‐cell proteomics experiments.

Single analytical runs of isobaric labeling experiments in bulk contain few missing values, but this is no longer the case for high carrier level samples (i.e., SCoPE) analyzed at single‐cell level. At a carrier ratio of 1:100 approximately 70% of all quantitative channels have no detectable signal.^59^ Through multi‐batch SCoPE2 experiments the authors report that only 10% of their missing data can be accounted to too low reporter ion intensity and stochastic MS2 sampling results in about 70%.^13^ Within the combination of nanoPOTS and SCoPE2 authors stringently filtered PSMs and proteins for a maximum of 40% missingness across all samples, dramatically reducing the dataset (i.e., 2331 proteins identified at <1% FDR to 1200 proteins with two unique peptides and > 40% quantified).^34^ IonQuant, for FDR controlled MBR in a re‐analysis of sparse single‐cell datasets resulted in less false positives when transferring identifications. This might become extremely effective for label‐free single‐cell proteomics studies to confidently transfer identifications from a higher input sample to single‐cell runs.^90^ Careful quality control and stringent filtering of scarce data is important in MS‐based proteomics and becomes critical for the biological conclusions based on single‐cell measurements.

## 7 OUTLOOK

The field of single‐cell proteomics is rapidly advancing, with the analysis of large oocytes to the profiling of single mammalian cells with over 1000 protein identifications.^16^ Advancements of every sample processing step are required to improve coverage of single‐cell profiles. First, miniaturization and automatization of sample processing and transfer to the LC substantially overcomes peptide losses.^27^, ^36^, ^37^, ^39^ Second, nanoliter flow rates and adequate column material enhance chromatographic performance (e.g., analyte concentration) and ionization efficiency.^45^, ^46^ Third, designated acquisition strategies in conjunction with fast, sensitive, and selective MS instrumentation improve data quality (i.e., reproducibility) of label‐free or multiplexed workflows.^65^, ^66^, ^67^, ^68^, ^72^, ^74^, ^75^ Fourth, appropriate data analysis or enrichment (e.g. spectral libraries) and postprocessing tools (e.g. MBR) maximize the information obtained from single‐cell measurements (^81^, ^82^, ^83^, ^87^, ^89^ ^,^ ^90^). Lastly, multiplexed or label‐free carrier‐based systems additionally overcome sample losses^11^, ^63^, ^64^ but must be extensively quality controlled and kept to a minimum to not impair accuracy.^59^ The diverse and increasing number of applications reported recently demonstrates the broad interest in proteome analysis with single‐cell resolution. As exemplified in this review several bottlenecks still have to be addressed and multi‐disciplinary efforts are needed to further drive analytical performance. We are confident that with further developments single‐cell proteomics will become a versatile tool contributing new insights to numerous research questions within the next years.

## CONFLICT OF INTEREST

The authors declare no conflict of interest.
